# Heuristic Evaluation on Mobile Interfaces: A New Checklist

**DOI:** 10.1155/2014/434326

**Published:** 2014-09-11

**Authors:** Rosa Yáñez Gómez, Daniel Cascado Caballero, José-Luis Sevillano

**Affiliations:** Department of Computer Technology and Architecture, ETS Ingenieria Informatica, Universidad de Sevilla, Avenida Reina Mercedes s/n. 41012 Seville, Spain

## Abstract

The rapid evolution and adoption of mobile devices raise new usability challenges, given their limitations (in screen size, battery life, etc.) as well as the specific requirements of this new interaction. Traditional evaluation techniques need to be adapted in order for these requirements to be met. Heuristic evaluation (HE), an Inspection Method based on evaluation conducted by experts over a real system or prototype, is based on checklists which are desktop-centred and do not adequately detect mobile-specific usability issues. In this paper, we propose a compilation of heuristic evaluation checklists taken from the existing bibliography but readapted to new mobile interfaces. Selecting and rearranging these heuristic guidelines offer a tool which works well not just for evaluation but also as a best-practices checklist. The result is a comprehensive checklist which is experimentally evaluated as a design tool. This experimental evaluation involved two software engineers without any specific knowledge about usability, a group of ten users who compared the usability of a first prototype designed without our heuristics, and a second one after applying the proposed checklist. The results of this experiment show the usefulness of the proposed checklist for avoiding usability gaps even with nontrained developers.

## 1. Introduction

*Usability is the extent to which a product can be used with effectiveness, efficiency, and satisfaction in a specified context of use* [1]. While usability evaluation of traditional browsers from pc environments—desktop or laptop—has been widely studied, mobile browsing from smartphones, touch phones, and tablets present new usability challenges [2]. Additionally, mobile browsing is becoming increasingly widespread as a way of accessing online information and communicating with other users. Specific usability evaluation techniques adapted to mobile browsing constitute an interesting and increasingly important study area.

*Usability evaluation assesses the ease of use of a website's functions and how well they enable users to perform their tasks efficiently* [3]. To carry out this evaluation, there are several usability evaluation techniques.

**Figure 1:**
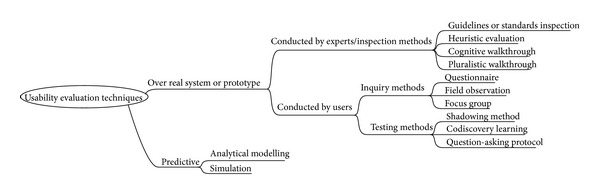
Classification of some usability evaluation techniques.

Usability evaluation techniques can be classified as shown in Figure 1 [4–8]. Over real systems or prototypes, the best alternatives are evaluations conducted by experts, also known as Inspection Methods, or evaluations involving users, which are divided into inquiry methods and testing methods depending on the methodology adopted. With a more academic focus, predictive evaluation offers some predictions over the usability of a potential and not-yet-existent prototype.

Heuristic evaluation (HE) is an inspection method based on evaluation over real system or prototype, conducted by experts. The term “expert” is used as opposed to “users” but in many cases evaluators do not need to be usability experts [9, 10]. In HE, experts check the accomplishment of a given heuristic checklist. Due to its nature, this inspection cannot be performed automatically.

HE, like other usability assurance techniques, has to take into account the fact that usability is not intrinsically objective in nature but is rather closely intertwined with an evaluator's personal interpretation of the artefact and his or her interaction with it [11]. But, evaluations can be designed to compensate for personal interpretation as much as possible.

Moreover, inspection methods are often criticized for only being able to detect a small number of problems in total together with a very high number of cosmetic ones [12]. But, HE presents several advantages over other techniques: its implementation is easy, fast, and cheap, and it is suitable for every life-cycle software phase and does not require previous planning [7]. Furthermore, it is not mandatory for evaluators to be usability experts [9, 10]. It is possible for engineers or technicians with basic usability knowledge to drive an evaluation. Furthermore, regarding the number of evaluators, Nielsen demonstrated empirically that between three and five experts should be enough [13].

Because of all these advantages, HE is a convenient usability evaluation method: the worst usability conflicts are detected at a low cost. But, traditional HE checklists are desktop-centred and do not properly detect mobile-specific usability issues [2].

In this study, we propose a heuristic guideline centred in mobile environments based on a review of previous literature. This mobile-specific heuristic guideline is not only an evaluation tool but also a compilation of recommended best-practices. It can guide the design of websites or applications oriented to mobile devices taking usability into account.

The following section describes the methods followed to define the mobile heuristic guideline. Then, Results and Discussion section is divided according to the steps defined in the methodology. We have included a brief discussion of the results for each task. The final sections include Conclusions and Future Work, Acknowledgments, and References.

## 2. Methods

To obtain a heuristic guideline centred in mobile environments and based on a review of previous literature, we will follow a six-step process.

1. A clear definition of the problem scope is necessary as a first step to define and classify the special characteristics of mobile interaction.
2. Next, we rearrange existing and well-known heuristics into a new compilation. We can reuse heuristic guidelines from the literature and adapt them to the new mobile paradigm because heuristic checklists derive from human behaviour, not technology [14]. This heuristics is general checks that must be accomplished in order to achieve a high level of usability.
3. After building this new classification of heuristics, we will develop a compilation of different proposed subheuristics. “Heuristic” in this paper refers to a global usability issue that must be evaluated or taken into account when designing. In contrast, the term “subheuristic” refers to specific guidelines items. The main difference between the two concepts lies in the level of expertise required of the evaluator and the abstraction level of the checklist. The resulting selection of subheuristics in this step takes into account some of the mobile devices restrictions presented in the first step. But, the result of this stage does not include many mobile specific questions, as they are not covered in traditional heuristic guidelines.
4. The fourth step in this work consists of enriching the list with mobile-specific subheuristics. This subheuristics is gleaned from mobile usability studies and best practices proposed in the literature.
5. One further step is required to homogenize the redaction and format of subheuristics in order to make it useful for nonexperts.
6. Finally, we conduct an evaluation of the usefulness of the tool as an aid in designing for mobile.

This process differs slightly from the methodology proposed by Rusu et al. [15], but we can subsume their phases when establishing new usability heuristics in our proposed method.

It is worth remarking that popular mobile operating systems are now providing usability guidelines [16, 17] which focus mainly on maintaining coherent interaction and presentation through applications over the whole platform. These guidelines could in some cases enrich certain aspects of our proposal, although we have opted to keep it essentially agnostic of specific platforms aesthetics or coherence-determined restrictions.

Additionally, interfaces for mobile are mainly divided into web access and native applications. We do not restrict our study to a specific kind of interface. Again, the goal is to elaborate a guideline which is independent of specific technologies. The interaction between users and mobile interfaces is similar regardless of the piece of software they are using.

## 3. Results and Discussion

### 3.1. Problem Scope Definition

Users are increasingly adopting mobile devices. According to statistics of Pew Internet & American Life Project [18], only in the USA 35% of adults own smartphones and 83% of adults own a cell phone of some kind. Additionally, 87% of smartphones owners access the Internet or email on their handheld—68% on a typical day. A further 25% say that they mostly go online using their smartphone, rather than a computer. This survey shows that phones operating on the Android platform are currently the most prevalent type, followed by iPhones and Blackberry devices.

Mobile usability involves different kind of devices, contexts, tasks, and users. The compilation of a new heuristic guideline needs a restriction and definition of the scope of the user-interface interaction.

Devices can be divided in three types [19]:

- feature phones: they are basic handsets with tiny screens and very limited keypads that are suitable mainly for dialing phone numbers;
- Smartphones: phones with midsized screens and full A–Z keypads;
- touch phones/touch tablets: devices with touch-sensitive screens that cover almost the entire front of the phone.

**Figure 2:**
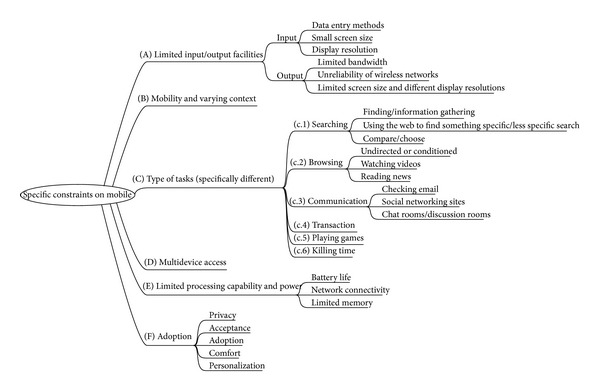
Specific constraints on mobile.

In our study, we have ruled out feature phones because the interaction and interface design are deeply restricted and they are gradually being abandoned by a wide range of users. We have also ruled out smartphones because interaction is dramatically different due to the keyboard and they are commonly constrained to enterprise use. This study focuses on the ubiquitous touch phones and touch tablets. In this work, we use the term “touch phones” to refer to both phones and tablets because they share a similar interaction paradigm and the constraints we describe in Figure 2.

Mobile interactions define a new paradigm characterized by a wide range of specific constraints: hardware limitations, context of use, and so forth. All these restrictions have been studied in the bibliography in order to define the issues that must be overcome to improve usability. According to the literature, the main constraints when designing for mobile devices are (Figure 2):

- *limited input/output facilities* [20–24]: these limitations are imposed by data entry methods, small screen size, display resolution, and available bandwidth, as well as unreliability of wireless networks;
- *mobility and varying context* [20–23]: traditional usability evaluation techniques have often relied on measures of task performance and task efficiency. Such evaluation approaches may not be directly applicable to the often unpredictable, rather opportunistic and relatively unstable mobile settings. Mobile devices use is on-the-run and interactions may take from a few seconds to minutes, being highly context-dependent. Environmental distractions have a significant effect on mobile interfaces usability and hence they need to be taken into account [25]. Context of use involves background noise, ongoing conversations, people passing by, and so on. Distractions can be auditory, visual, social, or caused by mobility.
- The context of use is so influent in the interaction that many authors propose testing in the field as indispensable to study interaction with mobile devices [26]. Laboratory testing seems incapable of completely assuring usability in this mobile paradigm. Some attempts to cover this contextual information have been documented in the literature: Po et al. [27] proposed inclusion of contextual information into the heuristic evaluation proposed by Nielsen and Molich [9]; Bertini et al. [28] discussed the capacity of expert-based techniques to capture contextual factors in mobile computing. Indeed, it is not trivial to integrate real-world setting/context into inspection methods which are conceived as laboratory testing techniques. In any case, laboratory testing and expert-based techniques are complementary. Both approaches can be used in preliminary analysis and design of prototypes but, even more in mobile than when dealing with old desktop interaction paradigms, they need to be complemented with users-based testing.
- *Type of Tasks*: in mobile environments, typical tasks are relatively different from traditional desktop devices. From the origins of mobile devices, concepts such as “personal space extension” [29] previewed new uses of mobile terminals.
- The literature has tended to classify mobile tasks on the basis of searching/browsing categories and also according to management of known information or new information. It is important to note that pre-2007 literature does not widely consider touch terminals which incorporate new tasks. Having taken all this into account, we can classify tasks as follows:
  - search [29–35]:
    - information gathering [33];
    - using the web to more or less specific search;
    - compare/choose [32];
  - browsing [30, 31, 33–35]:
    - undirected or conditioned browsing [31];
    - watching videos [14];
    - reading news [14];
  - communication [14, 33, 35]:
    - checking email [14];
    - social networking sites [14];
    - chat rooms/discussion rooms [33];
  - transaction [29, 33, 34]: although it is more common to use a mobile device to browse the web or to perform some shopping-related search than shopping in earnest [14].
  - playing games [14];
  - killing time [14].
- Some literature includes other kinds of task like “maintenance” [35] or “housekeeping” [33] that have not been included in our classification because the frequency of realization is too low and these kinds of tasks do not define new kinds of interactions.
- *Multidevice access*: user's familiarity with a web page [34] helps them to construct a mental model based on the structural organization of the information, such as visual cues, layout, and semantics. When a site is being designed for multidevice access, a major concern is to minimize user effort to reestablish the existing mental model. This new way of working around structured information that must be delivered through so many different interface restrictions has been studied as a new paradigm known as Responsive Design [36].
- *Limited processing capability and power* [21–24]: these limitations include battery life, network connectivity, download delays, and limited memory.
- *Adoption* [22]: adoption of mobile technology by users is based on perceived privacy, acceptance of technology, comfort, and capacity of personalization. Different levels of adoption determine different group of users interacting in a very different way with the interface. This may not seem to be a mobile-specific restriction but the wide variety of mobile devices, touchable or keyboard-based, with different sizes and presentation models, makes the range of users requiring different approaches much broader.

### 3.2. Rearrangement of Traditional Heuristics

**Figure 3:**
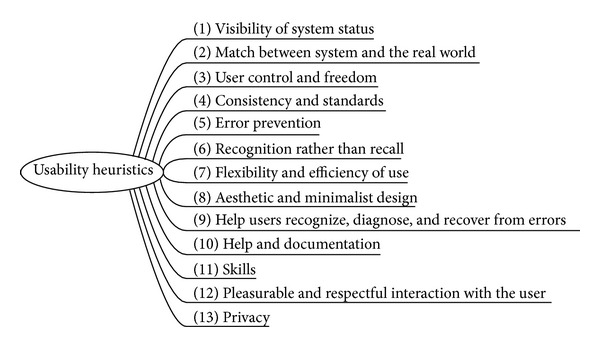
Proposed heuristic list.

The first rearrangement of traditional heuristics in this step is mainly based on the review of the literature by Torrente [7] where the author selected the most influent heuristics guidelines [9, 37–44]. This compilation gives a total of 9 heuristics guidelines consisting globally of 83 heuristics and 361 subheuristics. We need to rearrange this list of items into a new classification which is coherent to our purpose. In this step, we only take into account “heuristics” and no “subheuristics” and this gives us the heuristic list shown in Figure 3.

Our rearrangement focuses on literature coincidences (i.e., when the same concept or category is included in different works in the literature, perhaps under different names) and tries to propose a coherent, exhaustive, and complete framework of heuristics that could be used to arrange further identified subheuristics. Literature coincidences for each heuristics are as follows:

1. visibility of system status [9, 38, 44]: other bibliography references include this concept as “Track State” [41], “Give feed-back” [37], or “Feedback” [40];
2. match between system and the real world [9, 38];
3. user control and freedom [9, 38, 42, 43]: other bibliography references include this concept as “Support the user control” [37], “The feedback principle” [39], “Autonomy” [41], or “Visible Navigation” [41];
4. consistency and standards [9, 38, 41, 44] also cited as “Maintain Consistency” [37], “Structure Principle” [39], “Reuse Principle” [39], “Consistency” [40], or “Learnability” [41];
5. error prevention [9, 38, 40] also cited as “Tolerance Principle” [39];
6. recognition rather than recall [9, 38] also cited as “Reduce recent memory load for users” [37], “Structure principle” [39], “Reuse principle” [39], “Minimize the users' memory load” [40], or “Anticipation” [41];
7. flexibility and efficiency of use [9, 38] also cited as “Simplicity principle” [39] or “Look at the user's productivity not the computer's” [41];
8. aesthetic and minimalist design [9, 38];
9. help users recognize, diagnose, and recover from errors [9, 37, 38] also cited as “Good error messages” [40] or “In case of error, is the user clearly informed and not over-alarmed about what happened and how to solve the problem?” [42];
10. help and documentation [9, 38, 40, 42–44];
11. skills [38] also cited as “Prepare workarounds for frequent users” [37], “Shortcuts” [40], or “Readability” [41];
12. pleasurable and respectful interaction with the user [38] also cited as “Simplicity principle” [39], “Simple and natural dialog,” or “Speak the user's language” [40]: this point also includes any accessibility questions that could enrich usability allowing a more universal access, such as “Color blindness” [41];
13. privacy [38].

### 3.3. Compilation of Subheuristics from Traditional General Heuristic Checklists

As defined before, “heuristic” in this paper refers to a global usability issue which must be evaluated or taken into account when designing. In contrast, the term “subheuristic” refers to specific guidelines items. In this third step, we focus on locating subheuristics from the literature.

The first group of potential heuristics is the 361 subheuristics proposed in the 9 references selected by Torrente [7]. Among these sub-heuristics we exclude those that do not fit well with the previously described mobile constraints. For example, subheuristics referred to desktop data entry methods is obviously discarded. In contrast, this referring to screen use optimization is particularly relevant. Other discarded amounts of subheuristics include some proposed [38] with specific response times which do not apply in a mobile and varying context. We also discard coincidences between different authors proposals.

Thus, from a total of 361 amounts of subheuristics proposed by the 9 references [9, 37–44] selected by Torrente [7], in this study, we obtain a first selection of 158 subheuristics.

**Figure 4:**
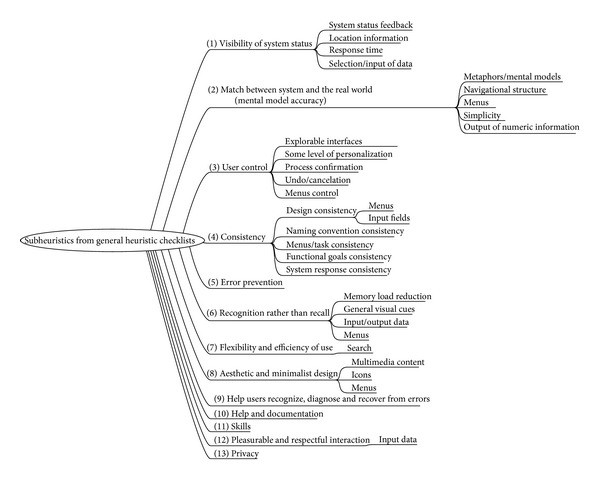
First framework for classification of detected subheuristics.

In order to maintain consistency in our classification, some subheuristics has been moved from their original heuristic parents, and new subcategories have been added so that semantically related amounts of subheuristics are grouped together. The final framework, shown in Figure 4, builds on that presented in the previous section.

It is also important to recall that at this stage, subheuristics redactions have been kept unchanged from their corresponding references. In the final compilation, these redactions will be modified in order to homogenize the whole guideline as we planned for step 4 in our methodology.

The final list of subheuristics is as follows:

(1) visibility of system status:

 system status feedback:
  1. is there some form of system feedback for every operator action? [38]
  2. if pop-up windows are used to display error messages, do they allow the user to see the field in error? [38]
  3. in multipage data entry screens, is each page labeled to show its relation to others? [38]
  4. are high informative contents placed in high hierarchy areas? [42]
 location information:
  (5) is the logo meaningful, identifiable, and sufficiently visible? [42]
  (6) is there any link to detailed information about the enterprise, website, webmaster … ? [42]
  (7) are there ways of contacting with the enterprise? [42]
  (8) in articles, news, reports … are the author, sources, dates, and review information shown clearly? [42]
 response times:
  (9) are response times appropriate for the users cognitive processing? [38]
  (10) are response times appropriate for the task? [38]
  (11) if there are observable delays (greater than fifteen seconds) in the system's response time, is the user kept informed of the system progress? [38]
  (12) latency reduction [41];
 Selection/input of data:
  (13) is there visual feedback in menus or dialog boxes about which choices are selectable? [38]. We will merge this statement with the following: “Do GUI menus make obvious which item has been selected?” [38], “Do GUI menus make obvious whether deselection is possible?” [38], “Is there visual feedback in menus or dialog boxes about which choice the cursor is on now?” [38], and “If multiple options can be selected in a menu or dialog box, is there visual feedback about which options are already selected?” [38]
  (14) is the current status of an icon clearly indicated? [38]
  (15) is there visual feedback when objects are selected or moved? [38]
  (16) are links recognizable? Is there any characterization according to the state (visited, active,…)? [42]

(2) match between system and the real world (Mental model accuracy):

 metaphors/mental models:
  (17) use of metaphors [41];
  (18) are icons concrete and familiar? [38]
  (19) if shape is used as a visual cue, does it match cultural conventions? [38]
  (20) do the selected colours correspond to common expectations about color codes? [38]
 navigational structure:
  (21) if the site uses hierarchical structure, are depth and height balanced? [42]
  (22) navigation map [44], also known as site map or table of contents;
 menus:
  (23) are menu choices ordered in the most logical way, given the user, the item names, and the task variables? [38]
  (24) do menu choices fit logically into categories that have readily understood meanings? [38]
  (25) are menu titles parallel grammatically? [38]
  (26) in navigation menus, are the number of items and terms by item controlled to avoid memory overload? [42]
 simplicity:
  (27) do related and interdependent fields appear on the same screen? [38]
  (28) for question and answer interfaces, are questions stated in clear, simple language? [38]
  (29) is the language used the same target users speak? [42]. We will merge this statement with the following: “Is the menu-naming terminology consistent with the user's task domain?” [38]
  (30) is the language clear and concise? [42]. We will merge this statement with the following: “Does the command language employ user jargon and avoid computer jargon?” [38]
  (31) does the site follow the rule “1 paragraph = 1 idea”? [42]
 output of numeric information:
  (32) does the system automatically enter leading or trailing spaces to align decimal points? [38]
  (33) does the system automatically enter a dollar sign and decimal for monetary entries? [38]
  (34) does the system automatically enter commas in numeric values greater than 9999? [38]
  (35) are integers right-justified and real numbers decimal-aligned? [38]

(3) user control:

 explorable interfaces:
  (36) can users move forward and backward between fields or dialog box options? [38]
  (37) if the system has multipage data entry screens, can users move backward and forward among all the pages in the set? [38]
  (38) if the system uses a question and answer interface, can users go back to previous questions or skip forward to later questions? [38]
  (39) clearly marked exits [40];
  (40) is the general website structure user-oriented? [42]
  (41) is there any way to inform user about where they are and how to undo their navigation? [42]
 some level of personalization:
  (42) can users set their own system, session, file, and screen defaults? [38]
 process confirmation:
  (43) when a user's task is complete, does the system wait for a signal from the user before processing? [38]
  (44) are users prompted to confirm commands that have drastic, destructive consequences? [38]
 undo/cancelation:
  (45) can users easily reverse their actions? [38] Also found as “Do function keys that can cause serious consequences have an undo feature?” [38] and “Is there an “undo” function at the level of a single action, a data entry, and a complete group of actions?” [38]
  (46) can users cancel out of operations in progress? [38]
 menus control:
  (47) if the system has multiple menu levels, is there a mechanism that allows users to go back to previous menus? [38]
  (48) are menus broad (many items on a menu) rather than deep (many menu levels)? [38]
  (49) if users can go back to a previous menu, can they change their earlier menu choice? [38]

(4) consistency:

 designing consistency:
  (50) are attention-getting techniques used with care? [38]
  (51) intensity: two levels only [38];
  (52) color: up to four (additional colors for occasional use only) [38];
  (53) are there no more than four to seven colors, and are they far apart along the visible spectrum? [38]
  (54) sound: soft tones for regular positive feedback, harsh for rare critical conditions [38];
  (55) if the system has multipage data entry screens, do all pages have the same title? [38]
  (56) do online instructions appear in a consistent location across screens? [38]
  (57) have industry or company standards been established for menu design, and are they applied consistently on all menu screens in the system? [38]
  (58) are there no more than twelve to twenty icon types? [38]
  (59) has a heavy use of all uppercase letters on a screen been avoided? [38]
  (60) is there a consistent icon design scheme and stylistic treatment across the system? [38]
 menus:
  (61) are menu choice lists presented vertically? [38]
  (62) if “exit” is a menu choice, does it always appear at the bottom of the list? [38]
  (63) are menu titles either centered or left-justified? [38]
 input fields:
  (64) are field labels consistent from one data entry screen to another? [38]
  (65) do field labels appear to the left of single fields and above list fields? [38]
  (66) are field labels and fields distinguished typographically? [38]
 naming convention consistency:
  (67) is the structure of a data entry value consistent from screen to screen? [38]
  (68) are system objects named consistently across all prompts in the system? [38]
  (69) are user actions named consistently across all prompts in the system? [38]
 menu/task consistency:
  (70) are menu choice names consistent, both within each menu and across the system, in grammatical style and terminology? [38]
  (71) does the structure of menu choice names match their corresponding menu titles? [38]
  (72) does the menu structure match the task structure? [38]
  (73) when prompts imply a necessary action, are the words in the message consistent with that action? [38]
 functional goals consistency:
  (74) where are the website goals? Are they well defined? Do content and services delivered match these goals? [42]
  (75) does the look & feel correspond with goals, characteristics, contents and services of the website? [42]
  (76) is the website being updated frequently? [42]
 system response consistency:
  (77) is system response after clicking links predictable? [42]
  (78) are nowhere links avoided? [42]
  (79) are orphan pages avoided? [42]

(5) error prevention:

 
  (80) are menu choices logical, distinctive, and mutually exclusive? [38]
  (81) are data inputs case-blind whenever possible? [38]
  (82) does the system warn users if they are about to make a potentially serious error? [38]
  (83) do data entry screens and dialog boxes indicate the number of character spaces available in a field? [38]
  (84) do fields in data entry screens and dialog boxes contain default values when appropriate? [38]

(6) recognition rather than recall:

 memory load reduction:
  (85) high levels of concentration are not necessary and remembering information is not required: two to fifteen seconds [38];
  (86) are all data a user needs on display at each step in a transaction sequence? [38]
  (87) if users have to navigate between multiple screens, does the system use context labels, menu maps, and place markers as navigational aids? [38]
  (88) after the user completes an action (or group of actions), does the feedback indicate that the next group of actions can be started? [38]
  (89) are optional data entry fields clearly marked? [38]
  (90) do data entry screens and dialog boxes indicate when fields are optional? [38]
  (91) is page length controlled? [42]
 general visual cues:
  (92) for question and answer interfaces, are visual cues and white space used to distinguish questions, prompts, instructions, and user input? [38]
  (93) does the data display start in the upper-left corner of the screen? [42]
  (94) have prompts been formatted using white space, justification, and visual cues for easy scanning? [38]
  (95) do text areas have “breathing space” around them? [42]
  (96) are there “white” areas between informational objects for visual relaxation? [42]
  (97) does the system provide visibility; that is, by looking, can the user tell the state of the system and the alternatives for action? [38]
  (98) is size, boldface, underlining, colour, shading, or typography used to show relative quantity or importance of different screen items? [38]
  (99) is colour used in conjunction with some other redundant cue? [38]
  (100) is there good colour and brightness contrast between image and background colours? [38]
  (101) have light, bright, saturated colours been used to emphasize data and have darker, duller, and desaturated colours been used to deemphasize data? [38]
  (102) is the visual page space well used? [42]
 input/output data:
  (103) on data entry screens and dialog boxes, are dependent fields displayed only when necessary? [38]
  (104) are field labels close to fields, but separated by at least one space? [38]
 Menus
  (105) is the first word of each menu choice the most important? [38]
  (106) are inactive menu items grayed out or omitted? [38]
  (107) are there menu selection defaults? [38]
  (108) is there an obvious visual distinction made between “choose one” menu and “choose many” menus? [38]

(7) flexibility and efficiency of use:

 search:
  (109) is the searching box easily accessible? [42]
  (110) is the searching box easily recognizable? [42]
  (111) is there any advanced search option? [42]
  (112) are search results shown in a comprehensive manner to the user? [42]
  (113) is the box width appropriated? [42]
  (114) is the user assisted if the search results are impossible to calculate? [42]

(8) aesthetic and minimalist design:

 
  (115) Fitt's Law [41]: the time to acquire a target is a function of the distance to and size of the target;
  (116) is only (and all) information essential to decision making displayed on the screen? [38]
  (117) are field labels brief, familiar, and descriptive? [38]
  (118) are prompts expressed in the affirmative, and do they use the active voice? [38]
  (119) is layout clearly designed avoiding visual noise? [42]
 multimedia content:
  (120) does the use of images and multimedia content add value? [42]
  (121) are images well sized? Are they understandable? Is the resolution appropriate? [42]
  (122) are cyclical animations avoided? [42]
 icons:
  (123) has excessive detail in icon design been avoided? [38]
  (124) is each individual icon a harmonious member of a family of icons? [38]
  (125) does each icon stand out from its background? [38]
  (126) are all icons in a set visually and conceptually distinct? [38]
 menus:
  (127) is each lower-level menu choice associated with only one higher level menu? [38]
  (128) are menu titles brief, yet long enough to communicate? [38]

(9) help users recognize, diagnose and recover from errors;

(10) help and documentation:

 
  (129) are online instructions visually distinct? [38]
  (130) do the instructions follow the sequence of user actions? [38]
  (131) if menu choices are ambiguous, does the system provide additional explanatory information when an item is selected? [38]
  (132) if menu items are ambiguous, does the system provide additional explanatory information when an item is selected? [38]
  (133) is the help function visible, for example, a key labeled HELP or a special menu? [38, 42]
  (134) is the help system interface (navigation, presentation, and conversation) consistent with the navigation, presentation, and conversation interfaces of the application it supports? [38]
  (135) navigation: is information easy to find? [38]
  (136) presentation: is the visual layout well designed? [38]
  (137) conversation: is the information accurate, complete, and understandable? [38]
  (138) is the information relevant? ([38], Help and documentation) [42] It should be relevant in the following aspects [38]: goal-oriented (what can I do with this program?), descriptive (what is this thing for?), procedural (how do I do this task?), interpretive (why did that happen?), and navigational (where am I?);
  (139) is there context-sensitive help? [38, 42]
  (140) can the user change the level of detail available? [38]
  (141) can users easily switch between help and their work? [38]
  (142) is it easy to access and return from the help system? [38]
  (143) can users resume work where they left off after accessing help? [38]
  (144) if a FAQs section exists, are the selection and redaction of questions and answers correct? [42]

(11) skills:

 
  (145) do not use the word “default” in an application or service; replace it with “Standard,” “Use Customary Settings,” “Restore Initial Settings,” or some other more specific terms describing what will actually happen [41];
  (146) if the system supports both novice and expert users, are multiple levels of error message detail available? [38]
  (147) if the system supports both novice and expert users, are multiple levels of detail available? [38]
  (148) are users the initiators of actions rather than the responders? [38]
  (149) do the selected input device(s) match user capabilities? [38]
  (150) are important keys (e.g., ENTER, TAB) larger than other keys? [38]
  (151) does the system correctly anticipate and prompt for the user's probable next activity? [38]

(12) pleasurable and respectful interaction:

 
  (152) protect users' work [41], also as “For data entry screens with many fields or in which source documents may be incomplete, can users save a partially filled screen?” [38]
  (153) do the selected input device(s) match environmental constraints? [38]
  (154) are typing requirements minimal for question and answer interfaces? [38]
  (155) does the system complete unambiguous partial input on a data entry field? [38]

(13) privacy:

 
  (156) are protected areas completely inaccessible? [38]
  (157) can protected or confidential areas be accessed with certain passwords [38]
  (158) is there information about how personal data is protected and about contents copyright? [38]

### 3.4. Compilation of Mobile-Specific Subheuristics

**Figure 5:**
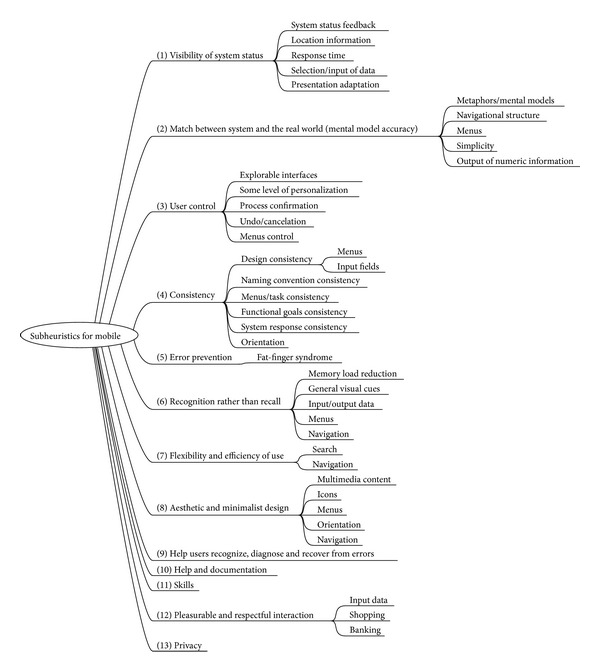
Second framework for classification of detected subheuristics.

The fourth step in this work is to enrich the list with mobile-specific subheuristics. The subheuristic list obtained in the previous section does not include many mobile specific questions because, as mentioned before, traditional heuristics does not usually cover these issues. New mobile-specific questions have been added into this list, taken from mobile usability studies and best practices that actually do not provide HE. Our approach allows us to include these new items into their corresponding categories, enriching the heuristic with mobile-specific issues. Some new categories had to be added to the original heuristic framework to include new mobile-specific subheuristics. The final framework is shown in Figure 5.

As we mentioned earlier, not all mobile devices have been considered; we discarded featured phones because they are rarely used for tasks other than phone calls and short message services (SMS) and they are gradually being abandoned apart from specific groups of users such as elderly or cognitively impaired people. We also discarded smartphones (phones with midsized screens and full A–Z keypads) because the interactivity with these devices is dramatically different from that of touch phones and they are commonly constrained to enterprise use. This study is centred in touch phones and tablets which are very popular nowadays and similar from a usability point of view.

This fourth step adds 72 new subheuristics to the compilation:

(1) visibility of system status:

 System status feedback:
  1. All the items on a list should go on the same page: if the items are text-only and if they are sorted in an order that matches the needs of the task [24];
  2. if a list of items can be sorted according to different criteria, provide the option to sort that list according to all those criteria [24];
  3. if a list contains items that belong to different categories, provide filters for users to narrow down the number of elements that they need to inspect [24];
  4. if the list contains only one item, take the user directly to that item [24];
  5. if the list contains items that download slowly (e.g., images), split the list into multiple pages and show just one page at a time [24];
  6. if an article spans several pages, use pagination at the bottom. Have a link to each individual page, rather than just to the previous and the next ones [24];
 location information:
  (7) whenever you have physical location information on your website, link it to a map and include a way of getting directions [24];
 response time:
  (8) splash screens too long [14];
  (9) download time [14]: “Progress bar is preferable” and “Alternative entertainment if download time is greater than 20 seconds”;
 selection/input of data:
  (10) low discoverability (active areas that do not look touchable): users do not know that something is touchable unless it looks as if it is [14];
  (11) swiping [14]: swiping is still less discoverable than most other ways of manipulating mobile content, so we recommended including a visible cue when people can swipe. And swipe ambiguity should be avoided: the same swipe gesture should not be used to mean different things on different areas of the same screen:
  (12) expandable menus should be used sparingly. Menu labels should clearly indicate that they expand to a set of options [14];
 presentation adaptation:
  (13) detect if users are coming to your site on a mobile phone and direct them to your mobile site [24];
  (14) include a link to your mobile site on your full site. It can direct mobile users who were not re-directed to your mobile site [24];
  (15) include a link to the full site on the mobile page [24];

(2) match between system and the real world:

 navigational structure:
  (16) too much navigation (TMN) [14];

(3) user control and freedom:

 explorable interfaces:
  (17) accidental activation (lack of back button) [24];
  (18) include navigation on the homepage of your mobile website [14];

(4) consistency and standards:

 orientation:
  (19) about constraining orientation: users tend to switch orientation when an impasse occurs and, if the application does not support them, their flow is going to be disrupted, and they are going to wonder why it is not working [14];
  (20) navigation (horizontal and vertical) must be consistent across orientations. Some applications use a different navigation direction in the two orientations; for instance, they use horizontal navigation in landscape and use vertical navigation in portrait [14];
  (21) inconsistent content across orientations [14]: “Same content,” “Keep location,” and “If a feature is only available in one orientation, inform users”;

(5) error prevention

 
  (22) accidental activation (lack of back button) [14];
 fat-finger syndrome:
  (23) touchable areas are too small [14]. Research has shown that the best target size for widgets is 1 cm × 1 cm for touch devices [14];
  (24) crowding targets: another fat-finger issue that we encountered frequently is placing targets too close to each other. When targets are placed too close to each other, users can easily hit the wrong one [14];
  (25) padding: although the visible part of the target may be small, there is some invisible target space that if a user hits that space, their tap will still count [14];
  (26) when several items are listed in columns, one on top of another (see the time example below), users expect to be able to hit anywhere in the row to select the target corresponding to that row. Whenever a design does not fulfil that expectation, it is disconcerting for users [14];
  (27) do not make users download software that is inappropriate for their phone [24];
  (28) JavaScript and Flash do not work on many phones; do not use them [24];

(6) recognition rather than recall:

 Memory load reduction:
  (29) the task flow should start with actions that are essential to the main task. Users should be able to start the task as soon as possible [14];
  (30) the controls that are related to a task should be grouped together and reflect the sequence of actions in the task [14];
 navigation:
  (31) use breadcrumbs on sites with a deep navigation structure (many navigation branches). Do not use breadcrumbs on sites with shallow navigation structures [24];

(7) Flexibility and efficiency of use:

 search:
  (32) a search box and navigation should be present on the homepage if your website is designed for smartphones and touch phones [24];
  (33) the length of the search box should be at least the size of the average search string. We recommend going for the largest possible size that will fit on the screen [24];
  (34) preserve search strings between searches. Use autocompletion and suggestions [24];
  (35) do not use several search boxes with different functionalities on the same page [24];
  (36) if the search returns zero results, offer some alternative searches or a link to the search results on the full page [24];
 navigation:
  (37) use links with good information scent (i.e., links which clearly indicate where they take the users) on your mobile pages [24];
  (38) use links to related content to help the user navigate more quickly between similar topics [24];

(8) aesthetic and minimalist design:

 
  (39) recognizable application icons to be found in the crowded list of applications [14];
 multimedia content:
  (40) getting rid of Flash content [14];
  (41) carousels [24]: avoid using animated carousels, but if they must be used, users should be able to control them;
  (42) do not use image sizes that are bigger than the screen. The entire image should be viewable with no scrolling [24];
  (43) for cases where customers are likely to need access to a higher resolution picture, initially display a screen-size picture and add a separate link to a higher resolution variant [24];
  (44) when you use thumbnails, make sure the user can distinguish what the picture is about [24];
  (45) use captions for images that are part of an article if their meaning is not clear from the context of the article [24];
  (46) do not use moving animation [24];
  (47) if you have videos on your site, offer a textual description of what the video is about. [24];
  (48) clicking on the thumbnail and clicking on the video title should both play the video [24];
  (49) indicate video length [24];
  (50) specify if the video cannot be played on the user's device [24];
  (51) use the whole screen surface to place information efficiently [14]: “Popovers for displaying information restricts size of frame where information will be shown” and “Small modal views present the same size constraints”;
 orientation:
  (52) desktop websites have a strong guideline to avoid horizontal scrolling. But for touch screens, horizontal swipes are often fine [19];
 navigation:
  (53) do not replicate a large number of persistent navigation options across all pages of a mobile site [24];

(9) Help users recognize, diagnose, and recover from errors:

 
  (54) To signal an input error in a form, mark the textbox that needs to be changed [24];

(10) help and documentation:

 
  (55) focus on one single feature at a time. Present only those instructions that are necessary for the user to get started [14];

(11) skills:

(12) pleasurable and respectful interaction:

 input data:
  (56) users dislike typing. Compute information for the users. For instance, ask only for the zip code and calculate state and town; possibly offer a list of towns if there are more under the same zip code [14];
  (57) be tolerant of typos and offer corrections. Do not make users type in complete information. For example, accept “123 Main” instead of “123 Main St.” [14];
  (58) save history and allow users to select previously typed information [14];
  (59) use defaults that make sense to the user [14];
  (60) If the application does not store any information that is sensitive (e.g., credit card), then the user should definitely be kept logged in (log out clearly presented) [14];
  (61) minimize the number of submissions (and clicks) that the user needs to go through in order to input information on your site [24];
  (62) When logging in must be done, use graphical passwords at least some of the time, to get around typing [24];
  (63) Do not ask people to register on a mobile phone; skipping registration should be the default option [24];
  (64) When logging in must be done, have an option that allows the user to see the password clearly [24];
 shopping:
  (65) when you present a list of products, use image thumbnails that are big enough for the user to get some information out of them [24];
  (66) on a product page, use an image size that fits the screen. Add a Link to a higher resolution image when the product requires closer inspection [24];
  (67) offer the option to email a product to a friend [24];
  (68) offer the option to save the product in a wish list [24];
  (69) on an e-commerce site, include salient links on the homepage to the following information: locations and opening hours (if applicable), shipping cost, phone number, order status, and occasion-based promotions or products [24];
 banking and transactions:
  (70) whenever users conduct transactions on the phone, allow them to save confirmation numbers for that transaction by emailing themselves. If the phone has an embedded screen-capture feature, show them how to take a picture of their screen [24];

(13) privacy:

 
  (71) for multiuser devices, avoid being permanently signed in on an application [14];
  (72) If the application does store credit card information, it should allow users to decide if they want to remain logged in [24]. Ideally, when the user opts to be kept logged in, he/she should get a message informing of the possible risks

### 3.5. Final New Mobile-Specific Heuristics

The final compilation of heuristics and subheuristics, which is shown in Appendix A (Supplementary Material available online at http://dx.doi.org/10.1155/2014/434326), gives a total of 13 heuristics and 230 subheuristics (158 + 72). In this final compilation, we have omitted intermediate classifications introduced during the discussion. Also, semantically related items have been merged into a single item following the most common presentation of heuristics guidelines in literature. Wording has been corrected to offer a homogeneous collection of heuristics questions.

This final mobile heuristics can be used as a tool to evaluate usability of mobile interfaces. In its current version, possible answers for the proposed questions are “yes/no/NA.” The number of “yes” answers provides a measure of the usability of the interface. Other approaches in the literature include more elaborates ratings that have to be agreed between evaluators [45].

### 3.6. Empirical Test of the New Mobile-Specific Heuristics

The goal of our test was to perform an evaluation of the usefulness of the proposed heuristics as a tool for designers and software engineers with no specific knowledge and experience of usability.

The use case design was as follows: two software engineers without any specific knowledge about usability were asked to design an interface for a tablet application having a functional description in a low-fidelity prototype designed for a desktop version of the application. Over their proposed interface design they used our heuristics as an evaluation and reflexion tool. In view of the results of the evaluation, they were asked to develop a new prototype. Finally, both interfaces were tested with a small group of users to compare their usability.

This empirical test of usefulness of the proposed usability list was divided into the following phases:

1. prototype 1: developing an interface prototype oriented to tablet access from a given PC-desktop low-fidelity functional design (prototype 1, P1);
2. HE of P1 using the proposed heuristics as the basis for an oriented discussion between designers;
3. prototype 2: evolution of P1 fixing usability gaps detected in phase 2 (prototype 2, P2);
4. Empirical comparison of prototypes: users' testing of P1 and P2.

#### 3.6.1. Prototype 1 Developing

The functional description of the desktop version used to build the prototypes evaluated in this testing was provided by Project PROCUR@ [46], an e-care and e-rehabilitation platform focused on neurodegenerative diseases patients, their carers, and health professionals. The project is based on the deployment of three social spaces for research and innovation (SSRI) [47] in the three validation scenarios: Parkinson's disease SSRI, acquired brain damage (ABD) SSRI, and Alzheimer's disease SSRI. The functional description corresponds to this latter SSRI and provides five low-fidelity interface descriptions from the point of view of five profiles: patients, relatives, doctors, caregivers, and sanitary personnel, respectively.

The subjects of these experiments were two software engineering students preparing their end of degree project. They had never been trained in usability but had knowledge about software life-cycles and design techniques. P1 was the result of a first tablet-interface adaptation without usability training. The tablet format was imposed because a bigger screen size is specially convenient for the target users (i.e., elderly people with low vision capability and motor control).

**Figure 6:**
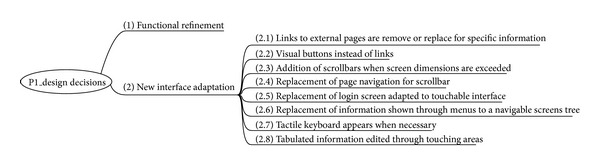
Design decisions in prototype 1.

This first adaptation included two main groups of changes: functional refinement and new interface adaptation. Functional refinement required changes that were not particularly relevant to this work. However, adaptations to the new interface involved decisions adopted by designers without knowledge of usability, guided only by their common sense. At a later stage, some of these decisions were confronted with the HE new tool and not all of them were maintained. These decisions are described in Figure 6.

**Figure 7:**
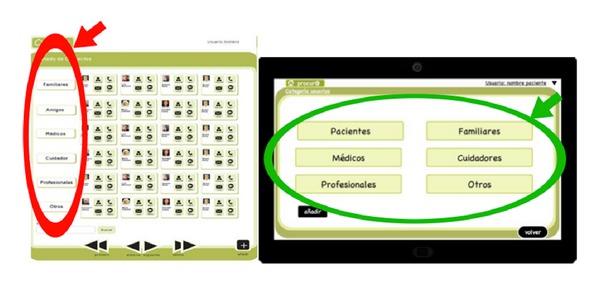
Prototype 1 from desktop version description.

Figure 7 shows an example of the interface change.

#### 3.6.2. Prototype 1: Heuristic Evaluation

Once Prototype 1 was designed, the next step was to evaluate its usability. The objective was not the evaluation itself but how the designers reflected on its usability.

When performing a HE using such tool, one has to make certain decisions about the scoring of each subheuristics. In this case, the experts were asked to use a ponderation which would allow the prioritization of heuristic item relevance for the specific evaluated interface. Experts are marked with values from 1 to 4: 1 for accomplished heuristic items, 2 for those corresponding to usability gaps, 3 for heuristic items which were not evaluable in the actual software life-cycle phase, and 4 for questions not applicable to the interface.

Applying a Delphi-based [48] approximation, both experts were asked to independently evaluate the interface using the list. Afterwards, the results of the evaluations were confronted and the experts had to agree in the case of items with different scorings.

In the independent evaluation, the level of coincidence of the experts was moderate and in the final HE scoring, where both experts agreed, the results were as follows: 68 items scored 1, 33 items scored 2, 41 scored 3, and 98 scored 4. This final result established a huge number of items as “not applicable.” This may have been because the heuristics was intended to be as general as possible, not focusing on any specific kind of application, and it therefore included an exhaustive list of checks.

The most important result from this evaluation was that experts were forced to reflect on each item in the heuristic guideline. For each not accomplished question, they learnt which usability gaps had to be avoided in the interface design. This learning provided a wider knowledge background when it came to designing next prototype.

#### 3.6.3. Prototype 2 Building

Prototype 2 was not only a series of modifications to Prototype 1 but also a complete revision of the whole interface concept. This global reflexion was guided by the expert discussion from the previous section.

**Figure 8:**
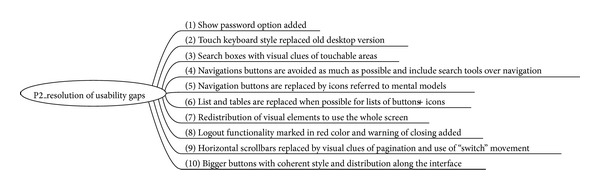
Prototype 2 main changes.

**Figure 9:**
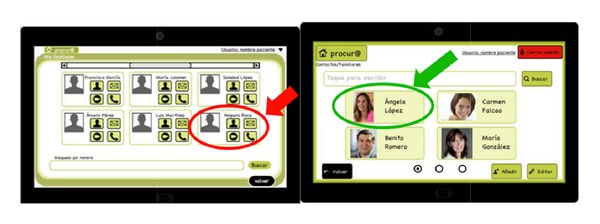
Prototype 2 global concept changes from the first version.

The most specific changes which fix detected usability gaps are shown in Figure 8 but, as mentioned, the overall appearance and design have changed dramatically (Figure 9).

### 3.7. Empirical Comparison of Prototypes

The empirical comparison of the two prototypes was intended to evaluate whether P2 designed using the proposed HE tool was better in any way than P1.

This empirical study involved users so the experiment had to be designed carefully to obtain valid results. The approach included a test design, a pilot phase to check the test design, the execution of the test itself, and a phase to analyze the collected data.

Several decisions were taken in the design phase.

1. Wizard of Oz [49] (WO) was chosen as the evaluation technique because the prototypes are developed on paper and are well suited to presentation through human intervention to the users.
2. To develop WO technique, users were asked to perform a task-guided interaction. The experts selected three functional tasks that users had to carry out interacting with the interface. The tasks were representative enough to be useful in this test. They were briefly described to the users so that they were able to accomplish them by exploring the interface without step-by-step guides.
3. Ten users were selected with the characteristics shown in Table 1.
4. The experimentation adopted an inner group [50] design: half of the users interacted with P1 first and the other half with P2 first. This was to avoid as much as possible correlations due to learning of the interfaces.
5. Lastly, users were asked to give feedback about their overall feelings about each interface to provide us with some conclusions related to user experience beyond usability.

The pilot phase consisted of a simulation of the final experiment using two dummy users. This phase was very useful for consolidating of task description and helping to improve Wizard's skills managing prototypes and for the whole experiment.

Final test execution detected 6 serious usability gaps in P1 and 3 serious gaps in P2 (which are also included in the first 6) as can be seen in Table 2. When asked about general satisfaction, 100% of users stated they were more satisfied with P2 prototype interaction.

## 4. Conclusions and Future Work

In this paper, we have presented a compilation of heuristic evaluation checklists readapted to mobile interfaces. We started our work by reusing heuristics from desktop heuristics evaluation checklists, which is allowed because “heuristic checklists change very slowly because they derive from human behaviour, not technology” [14]. In fact, in the final proposal of this work, the amount of reused heuristics from the literature is 69% of the total proposed subheuristics. The rest are best-practices and recommendations for mobile interfaces not initially conceived as part of a usability tool.

In the final collection of 13 heuristics, the most influent author is Nielsen [9, 37–44]. While it is not a long list of heuristics, it is exhaustive enough to be a useful categorization for further research. However, in our work, Nielsen's heuristics has been rearranged taking into account other proposals in the literature which emphasize concepts such as skills adaptation and pleasurable and respectful interaction with the user and privacy, elevating them to the category of heuristic item.

The added mobile-specific subheuristics in this proposal focus specifically on overcoming specific constraints on mobile such as limitations in input/output, limited processing capabilities, and power. Additionally, it focuses on favouring usual tasks in mobile and issues related to the adoption of this kind of devices (privacy, acceptance, comfort, personalization…).

The main original contributions of our work include (a) rearrangement of existing desktop heuristics into a new compilation, including detailed subheuristics, adapted to the new mobile paradigm; (b) enriching the list with mobile-specific subheuristics, mainly taken from mobile usability studies and best-practices proposed in the literature; (c) homogenization of the redaction and format of subheuristics in order to make it a useful and comprehensive tool for nonexperts; and (d) user-evaluation of the usefulness of the tool as an aid in designing for mobile.

Future work includes mobility and varying context and multidevice access, constraints that are not considered with enough detail in this work. Indeed, these two questions constitute specific areas of study. The typical mobility and varying context of this kind of devices highlight the limitations of laboratory testing: to fully test mobile interfaces, some field-testing is required. Multidevice access questions deal with Responsive Design [36], a discipline that manages access to a given source of information from different devices in a coherent and comprehensive manner.

Regarding rating, in this study, no weighting for categories was established. We mentioned the nonnegligible amount of items scored as nonapplicable in our experiment. Weighting specific categories or subsets of subheuristics according to the kind of application being evaluated represents a highly interesting area for future work and one which is closely related to certain advances in the work of Torrente [7].

The heuristic checklist we have proposed needs to be thoroughly validated in future research in relation to different aspects. The preliminary test and results obtained in this work appear to indicate that the proposed HE guideline is a useful tool for engineers, designers, and technicians with no specific knowledge in usability. A first hypothesis to explain this result is that more specific heuristic guidelines named subheuristics in this work are easier to manage for nonexpert evaluators. The specificity of the items collected in the tool means that it can be used as a reference guide to help conceive more usable interfaces and not just as a reactive evaluation tool for existing prototypes. Future work should look into this to confirm this partial result.

Furthermore, other aspects related to the suitability of this guideline need to be validated. For instance, an experts-guided review could evaluate the completion, coherence, and adequacy of the heuristic checklist. This review could be carried out through questionnaires, experts panels, or some kind of Delphi-based surveys [48]. Another highly interesting question is the empirical comparison of general heuristics and this mobile-specific heuristics when analysing mobile interfaces.

## Supplementary Material

The final compilation of heuristics and subheuristics gives a total of 13 amounts of heuristics and 230 amounts of subheuristics (158 + 72). In this final compilation, we have omitted intermediate classifications introduced during the discussion. Also, semantically related items have been merged into a single item following the most common presentation of heuristics guidelines in the literature. Wording has been corrected to offer a homogeneous collection of heuristics questions.
This final mobile heuristics can be used as a tool to evaluate usability of mobile interfaces. In its current version, possible answers for the proposed questions are "yes/no/NA." The number of “yes” answers provides a measure of the usability of the interface.

## Figures and Tables

**Table 1:** Users of the experiment.

	Gender	Age	Kind of mobile devices they are used to	Adoption of technology
USER 1	M	40–50	Touch phone	Basic
USER 2	F	40–50	Smartphone	None
USER 3	M	40–50	Smartphone	None
USER 4	F	40–50	Touch phone	Basic
USER 5	F	40–50	Touch phone	Basic
USER 6	F	40–50	Touch phone	Basic
USER 7	M	40–50	Touch phone	Basic
USER 8	F	40–50	Touch phone	Basic
USER 9	M	50–60	None	None
USER 10	F	50–60	Touch phone	Basic

**Table 2:** Results of empirical user-based evaluation of prototypes.

	Prototype 1. Usability gaps	Prototype 2. Usability gaps	Description
1	Authentication method inappropriate for the targeted users	Authentication method inappropriate for the targeted users	The boxes “user” and “password” should appear independently
2	Information screen confusing	Information screen confusing	It was maintained because the functional description included it
3	Chatting function not localizable	Returning to main menu not localizable	Even after changing the graphical clue
4	Personal profile function not localizable		
5	Returning to main menu not localizable		
6	Close session function not localizable		
