# Supplementary material for: Heuristic Evaluation on Mobile Interfaces: A New Checklist
Source: ScientificWorldJournal. 2014 Sep 11;2014:434326. doi: 10.1155/2014/434326 (PMC4177852; doi:10.1155/2014/434326)
Supplement: Supplementary file 1 — The final compilation of heuristics and subheuristics gives a total of 13 amounts of heuristics and 230 amounts of subheuristics (158 + 72). In this final compilation, we have omitted intermediate classifications introduced during the discussion. Also, semantically related items have been merged into a single item following the most common presentation of heuristics guidelines in the literature. Wording has been corrected to offer a homogeneous collection of heuristics questions. This final mobile heuristics can be used as a tool to evaluate usability of mobile interfaces. In its current version, possible answers for the proposed questions are "yes/no/NA." The number of “yes” answers provides a measure of the usability of the interface. [file 434326.f1.pdf]

Appendix A_Mobile-specific usability heuristic checklist

This final compilation of heuristics can be used as a tool to evaluate usability of mobile interfaces. In its current version, possible answers for the proposed questions are "yes/no/NA". The number of "yes" answers provides a measure of the usability of the interface. Other approaches include more elaborates ratings that have to be agreed between evaluators.

(1) Visibility of system status

System status feedback

1. Is there some form of system feedback for every operator action?
2. If pop-up windows are used to display error messages, do they allow the user to see the field in error?
3. In multipage data entry screens, is each page labeled to show its relation to others?
4. Are high informative contents placed in high hierarchy areas?
5. Do all the items on a list are on the same page? Are they sorted in an order that matches the needs of the task?
6. If a list of items can be sorted according to different criteria, does it provide the option to sort them according to all those criteria?
7. If a list contains items that belong to different categories, are there filters for users to narrow down the number of elements that they need to inspect?
8. If the list contains only one item, is the user taken directly to that item?
9. If the list contains items that download slowly (e.g., images), is the list split into multiple pages to show just one page at a time?
10. For articles which spans several pages, is pagination shown at the bottom? Is there a link to each individual page, rather than just to the previous and the next ones?

Location information

11. Is the logo meaningful, identifiable and sufficiently visible?
12. Is there any link to detailed information about the enterprise, web site, webmaster...?
13. Are there ways of contacting with the enterprise?
14. In articles, news, reports... Are the author, sources, dates and review information shown clearly?
15. For physical location information on the website, is a link to a map provided and the directions clearly accessible?

Response Times

16. Are response times appropriate for the users cognitive processing?
17. Are response times appropriate for the task?
18. If there are observable delays (greater than fifteen seconds) in the system's response time, is the user kept informed of the system progress?
19. Is latency sufficiently reduced?
20. Are splash screens too long avoided?
21. If download time is greater than 20 seconds, are progress bar offered instead of non-informative download screens?

Selection/input of data

22. Is there visual feedback in menus or dialog boxes about which choices are selectable?
23. Is the current status of an icon clearly indicated?
24. Is there visual feedback when objects are selected or moved?
25. Are links recognizable? Is there any characterization according to the state (visited, active,...)?
26. Are low discoverable areas as touch buttons well identifiable?
27. When swiping gesture is possible, is a visible clue offered to users? Is swiping used with a unique meaning in the same screen?
28. Are expandable menus used sparingly? Do menu labels clearly indicate that they expand to a set of options?

Presentation Adaptation

29. When users access to the site from a mobile phone, are they directed to the mobile version of the site?
30. Is a link to the mobile site provided on the desktop version of site?
31. Is a link to the full site included on the mobile page?

(2) Match between system and the real world (Mental model accuracy)

Metaphors/Mental models

32. Are metaphors properly used as visual clues?
33. Are icons concrete and familiar?
34. If shape is used as a visual cue, does it match cultural conventions?
35. Do the selected colours correspond to common expectations about colour codes?

Navigational Structure

36. If the site uses hierarchical structure, are depth and height balanced?
37. Is a navigation map or table of contents included on the site?
38. Is too much navigation avoided?

Menus

39. Are menu choices ordered in the most logical way, given the user, the item names, and the task variables?
40. Do menu choices fit logically into categories that have readily understood meanings?
41. Are menu titles parallel grammatically?
42. In navigation menus, are the number of items and terms by item controlled to avoid memory overload?

Simplicity

43. Do related and interdependent fields appear on the same screen?
44. For question and answer interfaces, are questions stated in clear, simple language?
45. Is the language used the same target users speak?
46. Is the language clear and concise?
47. Does the site follow the rule "1 paragraph=1 idea"?

Output of numeric information

48. Does the system automatically enter leading or trailing spaces to align decimal points?
49. Does the system automatically enter a dollar sign and decimal for monetary entries?
50. Does the system automatically enter commas in numeric values greater than 9999?
51. Are integers right-justified and real numbers decimal-aligned?

(3) User control and freedom

Explorable interfaces

52. Can users move forward and backward between fields or dialog box options?
53. If the system has multipage data entry screens, can users move backward and forward among all the pages in the set?
54. If the system uses a question and answer interface, can users go back to previous questions or skip forward to later questions?
55. Are exits clearly marked?
56. Is the general web site structure user-oriented?
57. Is there any way to inform user about where they are and how to undo their navigation?
58. Is accidental activation avoided or foreseen (a back button is offered)?
59. In mobile websites, is navigation on the homepage provided?

Some level of personalization

60. Can users set their own system, session, file, and screen defaults?

Process confirmation

61. When a user's task is complete, does the system wait for a signal from the user before processing?
62. Are users prompted to confirm commands that have drastic, destructive consequences?

Undo/Cancelation

63. Can users easily reverse their actions?
64. Can users cancel out of operations in progress?

Menus control

65. If the system has multiple menu levels, is there a mechanism that allows users to go back to previous menus?
66. Are menus broad (many items on a menu) rather than deep (many menu levels)?
67. If users can go back to a previous menu, can they change their earlier menu choice?

(4) Consistency and standards

Orientation

68. Is constraining orientation avoided? (Users tend to switch orientation when an impasse occurs and, if the app doesn’t support them, their flow is going to be disrupted and they are going to wonder why it’s not working)
69. Is navigation (horizontal and vertical) consistent across orientations? (Some applications use a different navigation direction in the two orientations; for instance, they use horizontal navigation in landscape and use vertical navigation in portrait).
70. Is content consistent across orientations?

Designing consistency

71. Are attention-getting techniques used with care?
72. Is intensity maintained in two levels only?
73. Is the number of colour used constrained up to four? Are additional colours saved for occasional use only?
74. Are the colour far apart along the visible spectrum?
75. Are soft tones used for regular positive feedback and harsh for rare critical conditions?
76. If the system has multipage data entry screens, do all pages have the same title?
77. Do on-line instructions appear in a consistent location across screens?
78. Have industry or company standards been established for menu design, and are they applied consistently on all menu screens in the system?
79. Are there no more than twelve to twenty icon types?
80. Has a heavy use of all uppercase letters on a screen been avoided?
81. Is there a consistent icon design scheme and stylistic treatment across the system?

Menus

82. Are menu choice lists presented vertically?

83. If "exit" is a menu choice, does it always appear at the bottom of the list?
84. Are menu titles either centred or left-justified?

Input fields

85. Are field labels consistent from one data entry screen to another?
86. Do field labels appear to the left of single fields and above list fields?
87. Are field labels and fields distinguished typographically?

Naming convention consistency

88. Is the structure of a data entry value consistent from screen to screen?
89. Are system objects named consistently across all prompts in the system?
90. Are user actions named consistently across all prompts in the system?

Menu/task consistency

91. Are menu choice names consistent, both within each menu and across the system, in grammatical style and terminology?
92. Does the structure of menu choice names match their corresponding menu titles?
93. Does the menu structure match the task structure?
94. When prompts imply a necessary action, are the words in the message consistent with that action?

Functional goals consistency

95. Where are the web site goals? Are they well defined? Do content and services delivered match these goals?
96. Does the look & feel correspond with goals, characteristics, contents and services of the web site?
97. Is the web site being updated frequently?

System response consistency

98. Is system response after clicking links predictable?
99. Are nowhere links avoided?
100. Are orphan pages avoided?

(5) Error prevention

101. Are menu choices logical, distinctive, and mutually exclusive?
102. Are data inputs case-blind whenever possible?
103. Does the system warn users if they are about to make a potentially serious error?
104. Do data entry screens and dialog boxes indicate the number of character spaces available in a field?
105. Do fields in data entry screens and dialog boxes contain default values when appropriate?
106. Is accidental activation avoided or foreseen (a back button is offered)?

Fat-finger syndrome

107. Are touchable areas sufficiently big? (Research has shown that the best target size for widgets is 1cmx1cm for touch devices)
108. Is crowding targets avoided? (When targets are placed too close to each other, users can easily hit the wrong one)
109. Although the visible part of the target may be small, is there some invisible target space that if a user hits that space, their tap will still count?
110. When several items are listed in columns, one on top of another, can users hit anywhere in the row to select the target corresponding to that row?
111. Is downloading of software that is inappropriate for the user phone avoided?
112. Are JavaScript and Flash use avoided?

(6) Recognition rather than recall

Memory load reduction

113. Are high levels of concentration not required and remembering information doesn't take more than two to fifteen seconds?
114. Are all data a user needs on display at each step in a transaction sequence?
115. If users have to navigate between multiple screens, does the system use context labels, menu maps, and place markers as navigational aids?
116. After the user completes an action (or group of actions), does the feedback indicate that the next group of actions can be started?
117. Are optional data entry fields clearly marked?
118. Do data entry screens and dialog boxes indicate when fields are optional?
119. Is page length controlled?
120. Do the task flow should start with actions that are essential to the main task? And can the users start the task as soon as possible?
121. Are the controls that are related to a task grouped together and reflect the sequence of actions in the task?

General visual cues

122. For question and answer interfaces, are visual cues and white space used to distinguish questions, prompts, instructions, and user input?
123. Does the data display start in the upper-left corner of the screen?
124. Have prompts been formatted using white space, justification, and visual cues for easy scanning?
125. Do text areas have "breathing space" around them?

126. Are there "white" areas between informational objects for visual relaxation?
127. Does the system provide visibility: that is, by looking, can the user tell the state of the system and the alternatives for action?
128. Are size, boldface, underlining, colour, shading, or typography used to show relative quantity or importance of different screen items?
129. Is colour used in conjunction with some other redundant cue?
130. Is there good colour and brightness contrast between image and background colours?
131. Have light, bright, saturated colours been used to emphasize data and have darker, duller, and desaturated colours been used to de-emphasize data?
132. Is the visual page space well used?

Input/output data

133. On data entry screens and dialog boxes, are dependent fields displayed only when necessary?
134. Are field labels close to fields, but separated by at least one space?

Menus

135. Is the first word of each menu choice the most important?
136. Are inactive menu items grayed out or omitted?
137. Are there menu selection defaults?
138. Is there an obvious visual distinction made between "choose one" menu and "choose many" menus?

Navigation

139. Is there a breadcrumbs on sites with a deep navigation structure (many navigation branches)? And, is it avoided on sites with shallow navigation structures?

(7) Flexibility and efficiency of use

Search

140. Is the searching box easily accessible?
141. Is the searching box easily recognizable?
142. Is there any advanced search option?
143. Are search results shown in a comprehensive manner to the user?
144. Is the box width appropriated?
145. Is the user assisted if the search results are impossible to calculate?
146. Is there a search box on the mobile site homepage?
147. Is the length of the search box at least the size of the average search string? Or better, is it the largest possible size that will fit on the screen?
148. Are search strings preserved between searches? Are there auto-completion and suggestions?
149. Are several search boxes with different functionalities on the same page avoided.
150. If the search returns zero results, is some alternative searches offered or a link to the search results on the full page?

Navigation

151. Are links with good information scent (that is, links which clearly indicate where they take the users)?
152. Are there links to related content to help the user navigate more quickly between similar topics?

(8) Aesthetic and minimalist design

153. Is the time to acquire a target is a function of the distance to and size of the target following Fitts Law?
154. Is only (and all) information essential to decision making displayed on the screen?
155. Are field labels brief, familiar, and descriptive?
156. Are prompts expressed in the affirmative, and do they use the active voice?
157. Is layout clearly designed avoiding visual noise?
158. Are application icons recognizable enough to be found in the crowded list of applications?

Multimedia content

159. Does the use of images and multimedia content add value?
160. Are images well sized? Are they understandable? Is the resolution appropriate?
161. Are cyclical animations avoided?
162. Is flash content avoided?
163. Is the use of animated carousels avoided? And if they exists, can users control them?
164. Are image sizes smaller than the screen? (The entire image should be viewable with no scrolling)
165. For cases where customers are likely to need access to a higher resolution picture, is a screen-size picture initially displayed and is there a separate link to a higher resolution variant?
166. When using thumbnails, can the user distinguish what the picture is about?
167. Does captions help to understand images meaning that are part of an article if their meaning is not clear from the context of the article?
168. Are moving animation avoided?
169. When using videos, is there a textual description of what the video is about?
170. Do clicking on the thumbnail and clicking on the video title both play the video?
171. Is video length indicated?
172. If the video cannot be played on the user’s device, is it a message shown with this information?

173. Is the whole screen surface used to place information efficiently (specially for popovers and modals)?

Icons

174. Has excessive detail in icon design been avoided?
175. Is each individual icon a harmonious member of a family of icons?
176. Does each icon stand out from its background?
177. Are all icons in a set visually and conceptually distinct?

Menus

178. Is each lower-level menu choice associated with only one higher level menu?
179. Are menu titles brief, yet long enough to communicate?

Orientation

180. Desktop websites have a strong guideline to avoid horizontal scrolling. But for touch screens, horizontal swipes are often fine. Is this option taken into account?

Navigation

181. Is the site designed to avoid a large number of persistent navigation options across all pages?

(9) Help users recognize, diagnose and recover from errors

182. When signalling an input error in a form, is the text box that needs to be changed specifically marked?

(10) Help and documentation

183. Are on-line instructions visually distinct?
184. Do the instructions follow the sequence of user actions?
185. If menu choices are ambiguous, does the system provide additional explanatory information when an item is selected?
186. If menu items are ambiguous, does the system provide additional explanatory information when an item is selected?
187. Is the help function visible; for example, a key labeled HELP or a special menu?
188. Is the help system interface (navigation, presentation, and conversation) consistent with the navigation, presentation, and conversation interfaces of the application it supports?
189. Navigation: Is information easy to find?
190. Presentation: Is the visual layout well designed?
191. Conversation: Is the information accurate, complete, and understandable?
192. Is the information relevant? It should be relevant in the following aspects: Goal-oriented (What can I do with this program?), Descriptive (What is this thing for?), Procedural (How do I do this task?), Interpretive (Why did that happen?) and Navigational (Where am I?).
193. Is there context-sensitive help?
194. Can the user change the level of detail available?
195. Can users easily switch between help and their work?
196. Is it easy to access and return from the help system?
197. Can users resume work where they left off after accessing help?
198. If a FAQs section exists, is the selection and redaction of questions and answers correct?
199. Is the design focused on one single feature at a time? (Only those instructions that are necessary for the user to get started should be presented at a time).

(11) Skills

200. Is the word "default" avoided and replaced with "Standard," "Use Customary Settings," "Restore Initial Settings," or some other more specific terms describing what will actually happen?
201. If the system supports both novice and expert users, are multiple levels of error message detail available?
202. If the system supports both novice and expert users, are multiple levels of detail available?
203. Are users the initiators of actions rather than the responders?
204. Do the selected input device(s) match user capabilities?
205. Are important keys (for example, ENTER , TAB) larger than other keys?
206. Does the system correctly anticipate and prompt for the user's probable next activity?

(12) Pleasurable and Respectful interaction

207. Is the users' work protected? For example, for data entry screens with many fields or in which source documents may be incomplete, can users save a partially filled screen?
208. Do the selected input device(s) match environmental constraints?
209. Are typing requirements minimal for question and answer interfaces?
210. Does the system complete unambiguous partial input on a data entry field?

Input data

211. Users dislike typing. Is information computed for the users? For instance, ask only for the zip code and calculate state and town; possibly offer a list of towns if there are more under the same zip code .
212. Is the input data tolerant of typos and offers corrections? (Don't make users type in complete information. For example, accept “123 Main” instead of “123 Main St.”)
213. Can users save history and select previously typed info?
214. Does default information make sense to the user?
215. If the app does not store any information that is sensitive (e.g. credit card), is the user kept logged in (with

log out clearly presented)?

216. Is the number of submissions (and clicks) minimized for the user going through in order to input information on the site?
217. When logging in must be done, are graphical passwords used at least some of the time, to get around typing?
218. Is registration not mandatory? Is skipping registration the default option?
219. When logging in must be done, is there an option that allows the user to see the password clearly?

Shopping

220. When a list of products is presented, are image thumbnails big enough for the user to get some information out of them?
221. On a product page, does image size fit the screen? Is there a link to a higher resolution image when the product requires closer inspection?
222. Is there the option to email a product to a friend?
223. Is there the option to save the product in a wish list?
224. On an e-commerce site, are salient links included on the homepage to the following information: — locations and opening hours (if applicable), — shipping cost, — phone number, — order status, and — occasion-based promotions or products?

Banking and Transactions

225. Whenever users conduct transactions on the phone, can they save confirmation numbers for that transaction by emailing themselves? If the phone has an embedded screen-capture feature, are there instructions about how to take a picture of their screen?

(13) Privacy

226. Are protected areas completely inaccessible?
227. Are protected or confidential areas only accessible with certain passwords?
228. Is there information about how personal data is protected and about contents copyright?
229. For multiuser devices: Is permanently signing in on an app avoided?
230. If the app does store credit card info, can users decide if they want to remain logged in? If the user opts to be kept logged in, he/she should get a message informing of the possible risks.
